# Double claw construct with hooks for proximal fixation in adolescent idiopathic scoliosis: a randomized controlled trial

**DOI:** 10.1007/s43390-025-01161-5

**Published:** 2025-08-28

**Authors:** J. J. M. Renkens, A. Willems, M. Reijman, P. de Baat, L. W. L. de Klerk, J. P. H. J. Rutges

**Affiliations:** 1 Department of Orthopedics and Sports Medicine, Erasmus MC Sophia Children’s Hospital, University Medical Centre, Dr. Molewaterplein40, 3015 GD Rotterdam, The Netherlands; 2 Department of Orthopaedic Surgery and Trauma, Catharina Hospital, Eindhoven, The Netherlands; 3 Department of Orthopedics, Sint Maartenskliniek, Ubbergen, The Netherlands

**Keywords:** Adolescent, Idiopathic, Scoliosis, Surgical technique

## Abstract

**Purpose:**

Proximal fixation in adolescent idiopathic scoliosis (AIS) surgery is a matter of discussion. All screw (AS) constructs provide better coronal correction than hybrid constructs, but high thoracic pedicle screw placement can be challenging. This study investigated whether an AS-construct provides better correction than a proximal double hook-claw (PH) construct.

**Methods:**

AIS patients undergoing posterior spinal fusion (PSF) were randomized to AS-construct or PH-construct from 2016 to 2020 with a two-year follow-up. Primary outcome is the main thoracic (MT) Cobb angle correction after two years. Secondary outcomes are thoracic kyphosis (TK), proximal junctional angle (PJA), proximal thoracic (PT) Cobb angle, SRS-22r, and complications after two years.

**Results:**

60 patients (30 AS, 30 PH) were included. No baseline differences were found. Preoperative MT Cobb angle was 62° ± 11° (AS) vs. 65° ± 12° (PH). There was no statistical difference in MT Cobb angle after two years: 25° ± 9° (AS) vs. 27° ± 7° (PH) (p = 0.247). No difference in PT Cobb angle was found: 20° ± 9° (AS) vs. 21° ± 9° (PH) and TK: 23° ± 9° (AS) vs. 22° ± 7° (PH). SRS-22r improved in both groups with no statistical difference: 3.9 ± 0.5 to 4.3 ± 0.5 (AS) vs. 3.7 ± 0.5 to 4.3 ± 0.5 (PH). There were 13 complications (ten patients) in AS group and 17 (13 patients) in PH group, including 1 major complication in each group (deep wound infection).

**Conclusion:**

AS-construct does not provide better coronal Cobb correction after two years after surgery. A PH-construct is a reliable and safe option for proximal fixation AIS patients. TRN: NTR-NL5552 (2016).

**Trial registration:**

Overview of medical research in the Netherlands (OMON): NL-OMON43852.

## Introduction

Surgical management of adolescent idiopathic scoliosis (AIS) aims to achieve fusion while maintaining correction and balance in the coronal and sagittal planes. Instrumentation techniques have evolved from all-hook constructs to hybrid systems (only screws at the distal end) and all-screw constructs. Although the use of pedicle screws at every level in posterior spinal fusion (PSF) is increasingly becoming standard practice, screw placement can be challenging, particularly in the proximal thoracic region and on the concave site of the main thoracic curve – due to smaller, more dysplastic pedicles and the close proximity of the spinal cord [1, 2]. These anatomical constraints can result in complications, including a screw malposition rate of up to 16% [3, 4].

All screw (AS)-constructs offer better coronal correction, fewer complications, and fewer reoperations [5, 6], while hybrid constructs are better at restoring thoracic kyphosis [7]. AS-constructs risk hypokyphosis, potentially leading to more proximal junctional kyphosis (PJK) [8, 9]. Helgeson et al. suggested that using transverse process hooks at the end of a screw construct reduces PJK [8]. Hooks in a claw construct at the end of a screw construct may ease the transition from rigid fixation to the more flexible thoracic spine, potentially reducing complications.

We conducted the first randomized controlled trial comparing two fixation techniques in the proximal thoracic region: a double claw-hook (PH) construct and an all-screw construct. The primary aim was to investigate if an AS-construct provides better correction than an PH-construct of the main thoracic (MT) Cobb angle two years post-surgery. Secondary aims included differences in loss of correction of MT, proximal thoracic (PT) Cobb angle correction and loss of correction, thoracic kyphosis, proximal junctional kyphosis, patient-reported outcome measures (PROMs), and complications in the two groups.

## Materials and methods

### Study design

The FIXIT trial is a single-center, single-blind, randomized controlled clinical trial evaluating whether the AS- construct is superior to the double PH- construct in correcting adolescent idiopathic scoliosis. Patients were recruited from February 2016 to June 2020. The local ethics committee approved the protocol, and all patients gave written informed consent. The trial was registered at the national trial register.

### Patients

Patients were recruited from the outpatient clinic at a tertiary referral hospital. Eligible patients were aged 10–20 years with AIS and a structural thoracic curve (Lenke type 1–4) requiring surgery. Exclusion criteria included neuromuscular or congenital scoliosis, planned combined anterior and posterior surgery, prior spinal surgery, intra-spinal pathology, or inability to speak or read Dutch. Patients and guardians received standardized oral and written information about the trial.

### Randomization and Masking

After informed consent and baseline measurements, patients were randomized 1:1 into two treatment groups by an independent researcher using a computer-generated list linked to a study identification number. Patients were blinded to the allocation. It was not possible to blind the researchers to the radiological measurements.

### Interventions

**Fig. 1:**
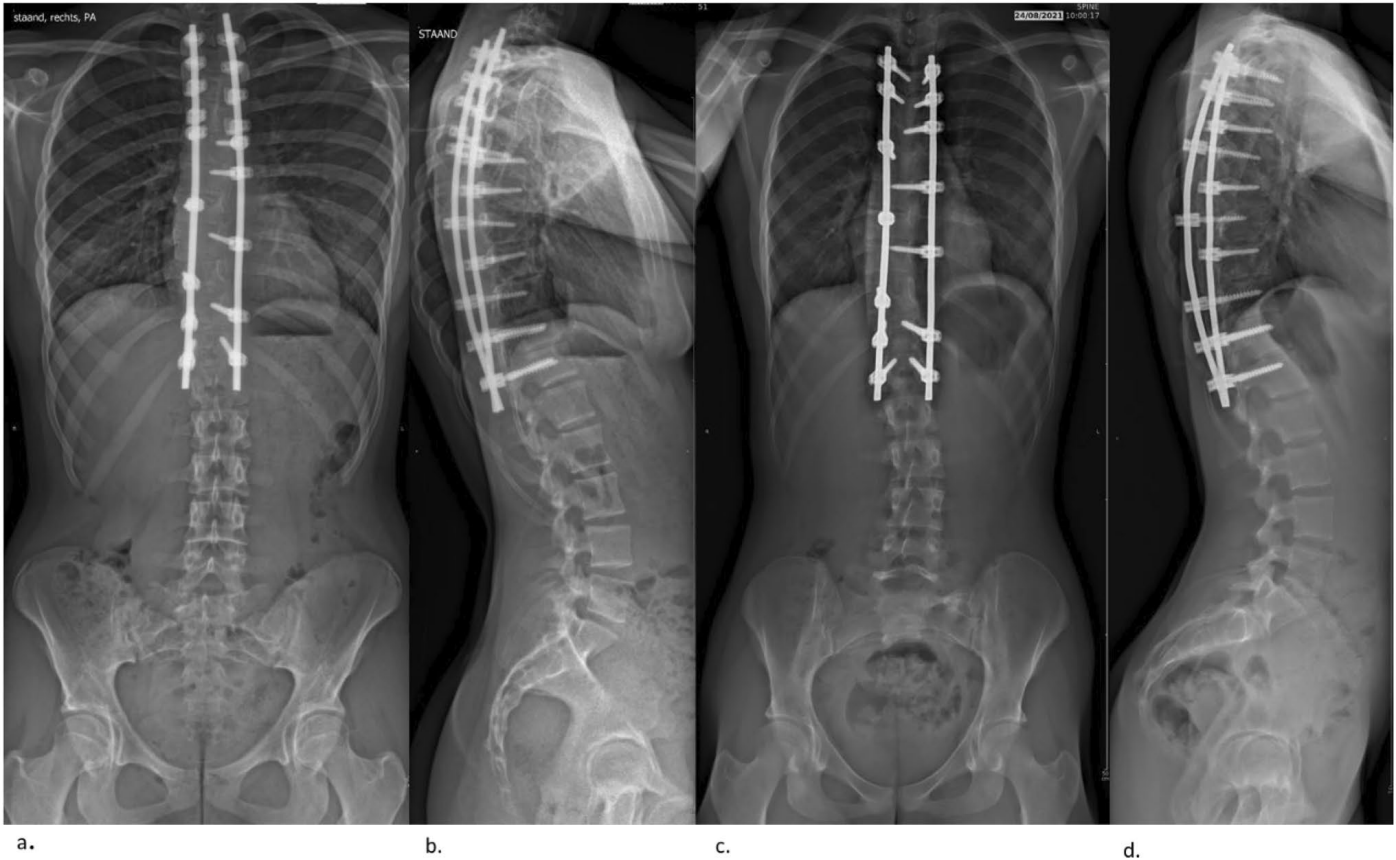
Two-year outcome radiographs. a. AP view claw-construct b. lateral view claw-construct c. AP view all-screw construct d. lateral view all-screw construct

The surgical team received the randomized allocation two to three days before surgery. The upper and lower instrumented levels were selected using the Lenke classification [10, 11]. Surgery was performed by two orthopedic surgeons from a pool of five, using a standard midline posterior approach. Screws were placed using a modified free-hand technique with fluoroscopy and electrical conductivity measurement or Dynamic Surgical Guidance (DSG) technology (PediGuard; SpineGuard, Vincennes, France). In the AS-group, uniplanar screws were used at every level. In the PH-construct group, transverse hooks were placed at the upper instrumented vertebra (UIV), one level below the UIV a transverse hook and a pedicle hook were placed and two levels below the UIV a pedicle hook was used (Fig. 1). A 5.5 mm cobalt-chromium rod was manually contoured and inserted at the proximal end on the convex side of the main thoracic (MT) curve. In the AS group, the proximal screws were first locked, while in the PH group, proximal fixation was achieved using hooks with compression-distraction. This was followed by sequential distal reduction of the rod into the screws using a cantilever method. Subsequently, a second over-bent rod was inserted on the concave side, using compression-distraction for the hooks in the PH-group and using segmental translation.

### Outcomes

The primary outcome was the between-group difference in MT coronal Cobb angles after two years. Secondary outcomes included coronal Cobb angle correction direct and one year postoperatively, correction of the PT curve, loss of correction of the MT and PT curve, sagittal Cobb angle correction, proximal junctional angle, vertebral rotation correction, patient-reported outcomes (SRS-22r), and complications. Patients were seen at baseline (preoperative), three months, one year, and two years postoperatively. X-rays were taken at these times, and questionnaires were completed on paper and transferred to SPSS. All X-rays were measured by one observer (JRe). A random sample of ten measurement for the MT, PT, bending, kyphosis and PJA angles was scored by a second observer (JRu) to assess interrater reliability.

### Sample size calculation

Based on a study by Kim et al. [12], a total of 50 patients (25 per group) was required to detect a significant difference in coronal Cobb angle correction postoperatively, with a power of 90% and alpha of 0.05. To accommodate a 20% dropout rate, the sample size was increased to 60 patients.

### Statistical analysis

Patients were analyzed according to their randomization. Statistical analysis was performed using IBM SPSS Statistics Version 28.0.1.0 (142). Linear regression analysis was used for the primary outcome, adjusted for baseline Cobb angle, randomized allocation, flexibility of the MT curve, and Lenke classification. Secondary outcomes were analyzed similarly. Linear mixed models were used to evaluate SRS-22r scores over the two-year follow-up, with randomized allocation, follow-up period, and their interaction as fixed factors. Model assumptions were checked and met.

Interrater reliability for the measured angles were analyzed by a two-way random-effect model with absolute agreement. Interrater reliability scores were interpreted according to the Koch-Landis method [13].

## Results

### Patients

**Fig. 2:**
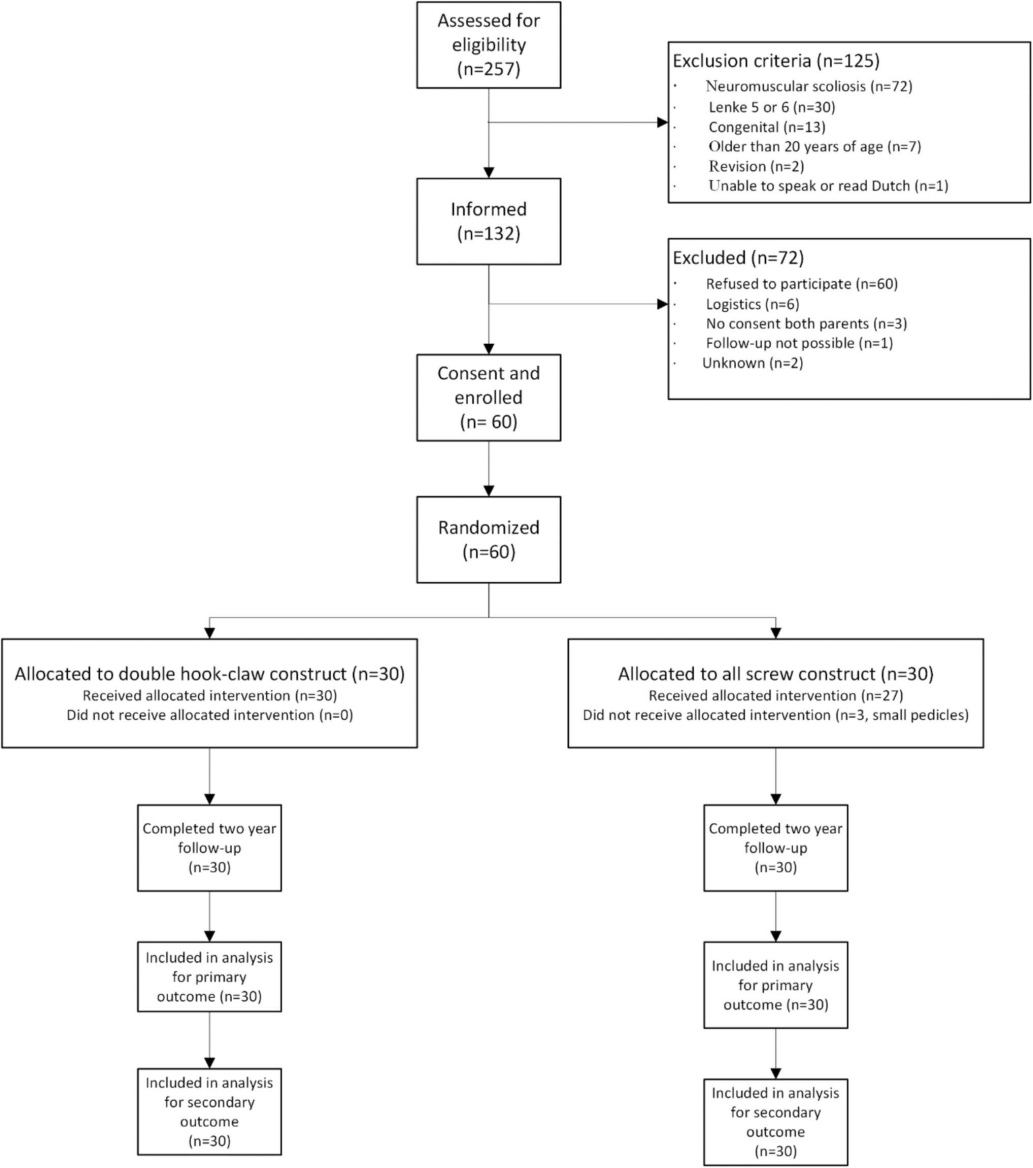
Flowchart

Sixty patients were included: thirty in both groups. One patient randomized to the AS- group received a PH- construct due to small pedicles seen on a preoperative CT scan. Two patients in the AS- group received a PH configuration on the right side of the proximal thoracic fixation (Fig. 2). All patients had a two-year follow-up with X-rays and PROMs.

Baseline characteristics are reported in Table 1. The mean preoperative MT coronal Cobb angle was 65° (SD 12°) in the PH-group and 62° (SD 11°) in the AS-group, with reducibility of 20% (standard deviation (SD) 15%) and 21% (SD 14%), respectively. Axial rotation was 17° (SD 7°) in the PH-group and 17° (SD 5°) in the AS-group.

**Table 1:** Study population characteristics. Data is presented as mean with standard deviation between parentheses, or reported otherwise. Implant density of the PT was calculated over Lenke 2 and 4 curves

	Claw-construct group (*n* = 30)	All-screw group (*n* = 30)
Age, years	15 (2) (range 12–19)	15 (2) (range 11–20)
Male sex, *n* (%)	8 (27)	6 (20)
BMI, kg/m^2^	22.2 (4.6)	21.1 (4.9)
Cobb angle main curve, degrees	65 (12)	62 (11)
Cobb angle proximal thoracic curve, degrees	33 (11)	31 (10)
Reducibility main thoracic curve, % (SD)	20 (15)	21 (14)
Lenke, *n* (%)		
1 & 3	16 (53)	18 (60)
2 & 4	14 (47)	12 (40)
Thoracal kyphosis, degrees	27 (15)	29 (15)
Duration of surgery, minutes	306 (48)	307 (58)
Blood loss, cc	550 (360)	511 (265)
Blood loss (%EBV)	14.1 (8.7)	13.6 (9.0)
Implant density		
Total	1.7 (0.1)	1.4 (.09)
Proximal thoracic	2.0 (0.3)	1.3 (0.3)
Main Thoracic	1.4(0.4)	1.2 (0.1)
Length of stay, days	6 (1)	6 (1)
Axial trunk rotation, degrees	17 (7)	17 (5)

### Primary outcome

**Fig. 3:**
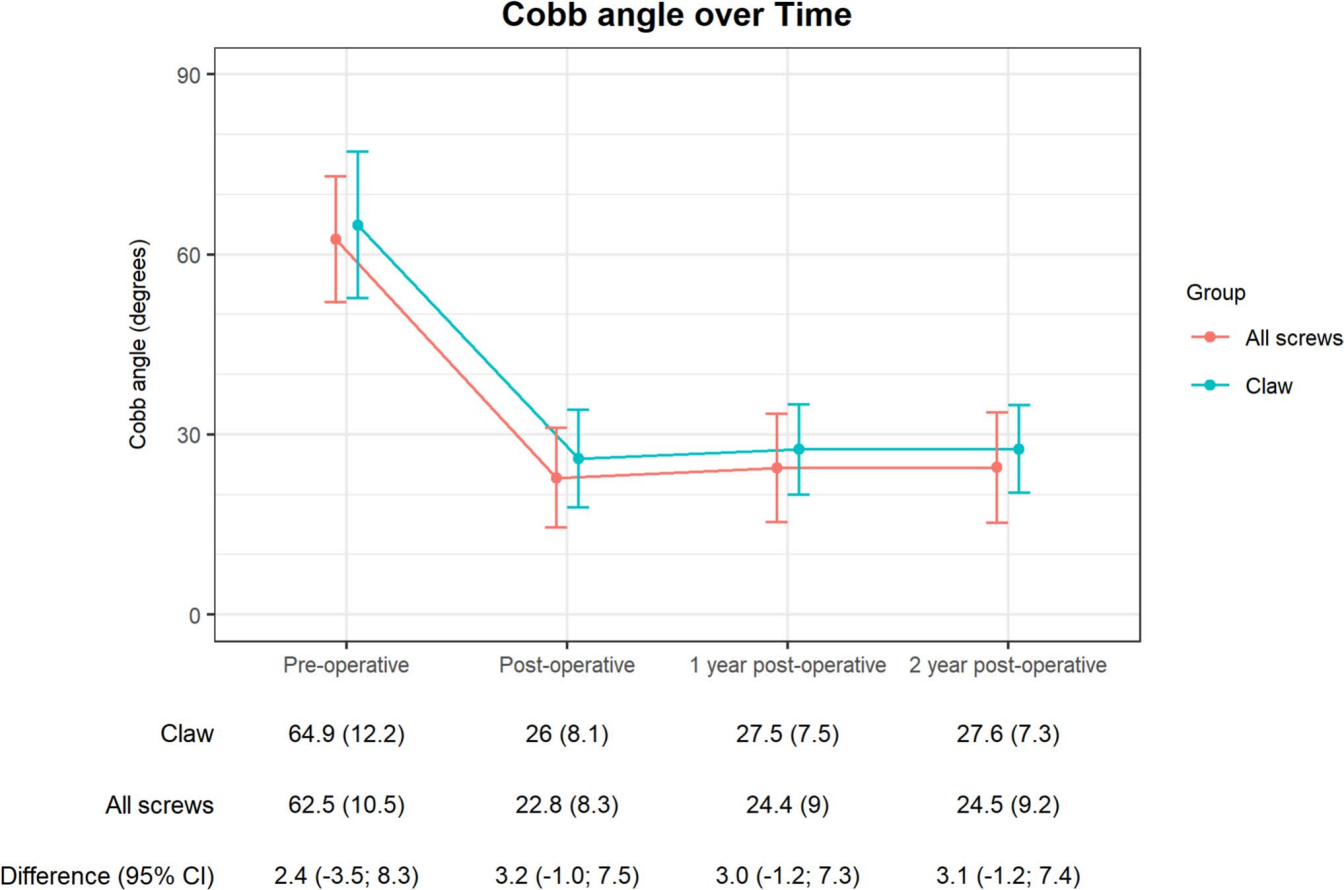
Primary outcome Cobb angle main thoracic curve in degrees

The coronal Cobb angle two years after surgery was 27.6° (SD 7.3°) in the PH-group and 24.5° (SD 9°) in the AS-group, with a between-group difference of 3.1° (95% confidence interval (CI) − 1.2 to 7.4, not significant) (Fig. 3).

### Secondary outcomes

The coronal Cobb angle increased significantly in both groups between direct postoperative and two years postoperative: 1.6° (95% CI 0.4–2.8, *p* < 0.001) in the PH- group and 1.7° (95% CI 0.2–3.2, *p* < 0.001) in the AS-group (Table 2). The between-group difference was not significant. PT curves decreased after surgery in both groups, with no significant difference two years postoperatively: 20.8° (SD 9.0°) in the PH-group and 19.9° (SD 9.2°) in the AS- group. Axial vertebral rotation improved, from 17° (SD 7°) to 11° (SD 6°) in the PH-group and from 17° (SD 5°) to 11° (SD 5°) in the AS-group, with no significant difference between groups.

**Table 2:** Secondary outcomes

	Baseline	Direct post-operative	1-Year post-operative	2-Year post-operative
Cobb angle proximal thoracic curve				
Proximal claw- hook construct group	32.6 (11.5)	19.5 (8.1)	20.7 (9.3)	20.8 (9.0)
All-screw group	31.4 (9.9)	18.9 (6.7)	19.8 (8.9)	19.9 (9.2)
Between group differences	1.2 (− 4.4; 6.7)	0.7 (− 3.2; 4.5)	0.8 (− 3.9; 5.5)	0.8 (− 3.9; 5.5)
Thoracic kyphosis				
Proximal claw-hook construct group	27.4 (15.0)	19.7 (8.5)	22.2 (7.1)	21.8 (6.8)
All-screw group	28.5 (15.0)	20.6 (8.3)	23.0 (9.2)	23.5 (9.0)
Between group differences	− 1.1 (− 9.0; 6.8)	− 1.0 (− 5.3; 3.4)	− 0.8 (− 5.1; 3.4)	− 1.7 (− 5.9; 2.4)
Proximal junctional angle				
Proximal claw- hook construct group		8.0 (5.4)		9.8 (6.3)
All-screw group		7.3 (4.0)		7.9 (5.0)
Between group differences		0.7 (− 1.7; 3.1)		1.9 (− 1.1; 5.0)
SRS total				
Proximal claw- hook construct group	3.7 (0.5)		4.1 (0.6)	4.3 (0.5)
All-screw group	3.9 (0.5)		4.3 (0.6)	4.3 (0.5)
Between group differences	− 0.12 (− 0.37; 0.13)		− 0.17 (− 0.47; 0.13)	− 0.00 (− 0.26; 0.26)

Data are presented as mean and standard deviation or 95% confidence interval between parentheses

In the sagittal plane, thoracic kyphosis decreased: from 27.4° (SD 15°) to 19.7° (SD 8.5°) direct postoperatively and 21.8° (SD 6.8°) at two years in the PH-group, and from 28.5° (SD 15.0°) to 20.6° (SD 8.3°) direct postoperatively and 23.5° (SD 9.0°) at two years in the AS-group. The between-group difference of 1.7° (95% CI − 5.9 to 2.4) at two years was not significant. Proximal junctional kyphosis, defined as the Cobb angle between the endplate of the UIV and the endplate two levels above the UIV, showed a between-group difference of 1.9° (95% CI − 1.1 to 5.0) two years postoperatively, which was not significant.

The total SRS-22r score improved significantly: from 3.7 (SD 0.5) to 4.3 (SD 0.5) in the PH-group and from 3.9 (SD 0.5) to 4.3 (SD 0.5) in the AS-group, with no significant between-group difference. All subdomains, except mental health, showed significant improvement with no significant between-group difference (Table 2 and Appendix).

### Complications

There was one serious adverse event (deep wound infection) in each group. In the PH-group, 13 patients had 16 adverse events and in the AS-group, ten patients had 12 adverse events (Table 3).

**Table 3:** Complication registration

	Proximal hook claw construct (*n* = 13/30)	All screw construct (*n* = 10/30)
Serious adverse event		
Deep wound infection	1	1
Adverse events		
Proximal junctional kyphosis	3	2
Pain	5	1
Shoulder imbalance	–	2
Instrumentation	3	2
Neurology (numbness chest)	–	2
Distal add-on	–	1
Medical	3	1
Wound (unsightly scar)	2	–
Coronal imbalance	–	1
Total complications	17	13

### Interrater reliability

The inter-rater-reliability was excellent for all groups (MT *k* = 0.99 95% CI 0.99–1.0, kyphosis *k* = 0.81 95% CI 0.29–0.95, bending *k* = 0.99 (95% CI 0.95–0.99 en PT *k* = 0.98 (95% CI 0.92–0.99), except for PJA (*k* = 0.76 95% CI 0.02–0.94).

## Discussion

This is the first single-blind randomized controlled trial comparing proximal hooks in a double claw construct to an all-screw construct in AIS surgery. The trial showed no significant difference between groups in main thoracic curve correction, implant-related complications, proximal curve correction, kyphosis, and patient satisfaction.

### Main curve correction

This study found no difference between a PH-construct and an AS-construct in correcting the MT Cobb angle at any time point. Literature reports mean Cobb angle correction of 52–73%, depending on the primary Cobb angle magnitude [14–19]. This is similar what we found in this study, namely a mean Cobb angle correction of 57–60%. The somewhat lower correction in our study can be explained by the relatively stiff scoliosis, with patient flexibility at 20–21%, which is lower than in other AIS studies [5]. The use of hooks or screws at the proximal end did not affect the MT Cobb angle correction.

### Complications

There was one implant-related complication in the PH- group and two screw-related complications in the AS-group. Ironically, the claw-construct complication was a misplaced blocking screw. No patient required revision surgery. Despite low reported complications, screw misplacement might be underreported. Hicks et al. noted a high rate of screw malposition in scoliosis surgery (up to 15.7%) using postoperative CT scans [4]. Bindels et al. found that 3D imaging during surgery improves screw positioning [20], though it is not widely available. While screw-related complications can be severe, they are rarely reported [4]. Hook-related complications are even rarer [21]. Long-term implications of malpositioned screws, such as chronic pain and loss of correction, can be severe [22, 23]. Despite potential underreporting, this study found no significant difference in implant-related complications, indicating both techniques are safe.

### Proximal curve correction

The best strategy for evaluating and correcting structural proximal thoracic curves remains to be elucidated [24, 25]. Correction may depend on the flexibility of the proximal curve and the fixation method of the proximal vertebrae. Our study found no difference in proximal thoracic curve correction between groups, averaging 36%, consistent with literature [19, 26, 27], even when including structural proximal curves (Lenke 2 and 4). This suggests that an AS-construct does not provide better correction than a PH-construct.

### Kyphosis

This study showed a significant decrease in thoracic kyphosis in both groups, averaging 28%. Loss of kyphosis was also noted by Lonner and Silvestre et al. [14, 28]. While hybrid hook constructs are associated with maintaining or restoring thoracic kyphosis [7], this was not observed for the PH-construct in our study. Proximal junctional kyphosis (PJK) increased slightly two years post-surgery, with three patients in the PH-group and two in the AS-group showing increases of more than ten degrees, none requiring revision surgery. Our results suggest no difference in PJK between the two fixation techniques. Literature shows conflicting results on anchor types at the UIV [8, 16, 19]. Helgeson et al. suggested hooks at the UIV could prevent PJK, but Pahys et al. found no difference between all-screws and hooks. This indicates PJK pathophysiology is multifactorial [29, 30], as confirmed by Ji et al.’s meta-analysis, which highlighted the significant contributions of Lenke classification and sagittal parameters to PJK.

### Construct stability

While primary correction is essential, maintaining correction stability over time is crucial. Hybrid constructs are prone to more loss of correction [14, 17]. Although the MT Cobb angle increased significantly (1.6° in PH- group and -1.7°in AS-group) over two years, this change is not clinically relevant. The PT Cobb angle showed no loss of correction in either group after two years. Kyphosis recovered significantly in both groups: − 2.1° (95% CI -− 3.6 to − 0.6, *p* = 0.004) in the PH- group and 2.7° (95% CI − 4.4 to − 0.9, *p* = 0.002) in the AS-group. Despite statistical significance, this recovery is not clinically relevant. Thus, a PH-construct is as stable as an AS-construct compared to hybrid constructs.

### Patient satisfaction (PROMs)

This study shows a significant improvement in health-related quality of life from preoperative to two years post-surgery (SRS-22r). Improvements were noted in total score and subdomains of pain, self-image, and satisfaction. Mens et al. reported similar SRS-22r total score improvements of 0.5 (SD 0.5), with comparable improvements in pain (0.6, SD 0.8) and self-image (1.1, SD 0.7) [31]. There was no difference between the two groups, indicating both techniques lead to satisfying patient experienced clinical outcomes.

### Limitations and strengths

The strength of this study is that it is the first randomized controlled trial comparing two proximal fixation techniques with no loss to follow-up after two years. However, it has some limitations. Primary the use of hooks in a claw construct is mainly described in hybrid constructs [32, 33] and minimally invasive bipolar constructs [34]. The specific proximal double hook-claw configuration used is not described in literature, limiting comparisons. Secondly, radiological assessment of the sagittal plane, especially in the high thoracic region, was challenging due to low-quality X-rays and overlapping structures. Additionally, 2D imaging was used, while 3D images provide more accurate information about spinal deformity [35, 36].

Finally, there were three cross-overs from the AS-group to the PH-group due to small proximal thoracic pedicles seen preoperatively. This may have led to more favorable outcomes in the AS-group with fewer complications, due to intention-to-treat analysis.

### Clinical implications

Proximal pedicle screw placement can be challenging in small, dysplastic pedicles in proximity to the spinal cord. 3D navigation or printed guides can assist but are expensive. A proximal double hook-claw offers a reliable option for proximal thoracic spine fixation in AIS patients.

## Conclusion

This study provides level 1 evidence that a proximal double hook-claw construct is not merely an alternative when pedicle screw placement is challenging—it represents a strategic and effective choice for proximal thoracic fixation. PH constructs offer strong, rigid stabilization and achieve comparable outcomes to all-screw constructs in terms of coronal and sagittal correction, patient satisfaction, and safety.

## Data Availability

Individual de-identified participant data that underlie the results reported in this paper (text, tables, figures and appendices) and the study protocol will be shared if requested. Data will be available beginning 12 months and ending 5 years following publication of this paper. Data will be available for researchers who provide a methodologically sound scientific proposal, which has been approved by an ethical committee. Proof of the latter should be provided. Analyses should achieve the aims as reported in the approved proposal. Proposals for data should be directed to: j.renkens@erasmusmc.nl.

## Appendix SRS-22r subdomains scores

**Table:**

Domain	Baseline	1-Year post-operative	2-Year post-operative
Function			
Group 1	4.3 (0.6)	4.4 (0.63)	4.6 (0.5)
Group 2	4.4 (0.5)	4.7 (0.4)	4.7 (0.4)
Between group differences	− 0.11 (− 0.41; 0.19)	− 0.30 (− 0.55; − 0.06)	− 0.08 (− 0.32; 0.16)
Pain			
Group 1	3.4 (0.8)	4.0 (0.9)	4.3 (0.7)
Group 2	3.7 (0.9)	4.1 (0.8)	4.3 (0.7)
Between group differences	− 0.30 (− 0.74; 0.15)	− 0.17 (− 0.61; 0.27)	− 0.09 (− 0.45; 0.28)
Self-image			
Group 1	3.3 (0.6)	4.0 (0.6)	4.2 (0.5)
Group 2	3.4 (0.6)	4.1 (0.7)	4.1 (0.7)
Between group differences	− 0.05 (− 0.34; 0.25)	− 0.10 (− 0.44; 0.23)	0.06 (− 0.28; 0.40)
Mental health			
Group 1	3.9 (0.8)	3.8 (0.7)	4.0 (0.6)
Group 2	4.0 (0.6)	4.1 (0.7)	4.0 (0.6)
Between group differences	− 0.07 (− 0.44; 0.30)	− 0.30 (− 0.67; 0.07)	− 0.01 (− 0.35; 0.32)
Satisfaction			
Group 1	4.1 (0.9)	4.6 (0.7)	4.8 (0.4)
Group 2	4.1 (0.9)	4.3 (0.9)	4.6 (0.7)
Between group differences	0.03 (− 0.43; 0.50)	0.270 (− 0.15; 0.68)	0.22 (− 0.08; 0.53)
